# Hyperbaric oxygen therapy as a complementary treatment in neuroblastoma — a narrative review

**DOI:** 10.3389/fonc.2023.1254322

**Published:** 2023-09-26

**Authors:** Diogo Alpuim Costa, J. Guilherme Gonçalves-Nobre, Mafalda Sampaio-Alves, Nuno Guerra, Joana Arana Ribeiro, Carla Espiney Amaro

**Affiliations:** ^1^ Hematology and Oncology Department, CUF Oncologia, Lisbon, Portugal; ^2^ Centro de Medicina Subaquática e Hiperbárica (CMSH), Portuguese Navy, Lisbon, Portugal; ^3^ Medical Oncology Department, Hospital de Cascais Dr. José de Almeida, Alcabideche, Portugal; ^4^ NOVA Medical School, Faculdade de Ciências Médicas da Universidade NOVA de Lisboa, Lisbon, Portugal; ^5^ Faculty of Medicine, University of Lisbon, Lisbon, Portugal; ^6^ Hospital Garcia de Orta (HGO), E.P.E., Almada, Portugal; ^7^ Instituto de Saúde Ambiental (ISAMB), Faculty of Medicine, University of Lisbon, Lisboa, Portugal; ^8^ Instituto de Medicina Preventiva & Saúde Pública (IMP&SP), Faculty of Medicine, University of Lisbon, Lisboa, Portugal; ^9^ PTSurg – Portuguese Surgical Research Collaborative, Lisboa, Portugal PTSurg – Portuguese Surgical Research Collaborative, Lisbon, Portugal; ^10^ Faculty of Medicine, University of Porto, Oporto, Portugal; ^11^ Pulmonology Department, Unidade Local de Saúde da Guarda, Guarda, Portugal

**Keywords:** neuroblastoma, treatment, therapy, iodine radioisotopes, iodine-131, radiopharmaceuticals, hyperbaric oxygenation, hyperbaric oxygen therapy

## Abstract

Neuroblastoma is the most frequently diagnosed cancer during the first year of life. This neoplasm originates from neural crest cells derived from the sympathetic nervous system, adrenal medulla, or paraspinal ganglia. The clinical presentation can vary from an asymptomatic mass to symptoms resulting from local invasion and/or spread of distant disease spread. The natural history of neuroblastoma is highly variable, ranging from relatively indolent biological behavior to a high-risk clinical phenotype with a dismal prognosis. Age, stage, and biological features are important prognostic risk stratification and treatment assignment prognostic factors. The multimodal therapy approach includes myeloablative chemotherapy, radiotherapy, immunotherapy, and aggressive surgical resection. Hyperbaric oxygen therapy (HBOT) has been proposed as a complementary measure to overcome tumor hypoxia, which is considered one of the hallmarks of this cancer treatment resistance. This article aims to review the relevant literature on the neuroblastoma pathophysiology, clinical presentation, and different biological and genetic profiles, and to discuss its management, focusing on HBOT.

## 1 Background

Although neuroblastoma is extremely rare in adults, it is the most frequently diagnosed cancer during the first year of life. Corresponding to more than 7% of neoplasms in patients younger than 15 years, it accounts for about 15% of pediatric cancer deaths. The age of presentation is between 18 and 22 months, with most cases diagnosed before 5 years of age. Despite being more frequent in Caucasian children, race does not seem to impact the prognosis (1–3).

The etiological factors are unknown due to the low prevalence of this disease. The potential role of maternal exposures during pregnancy, namely tobacco, alcohol, drugs and medication, chronic or infectious diseases, and vitamin supplements, has not yet been unequivocally established. While most neuroblastomas occur sporadically, about 1% of cases are hereditary by an autosomal dominant pattern with incomplete penetrance. These have distinctive characteristics: early appearance, multiple primary tumors, and good prognosis. In 5% of cases, there is an association with congenital diseases, namely von Recklinghausen’s disease, neurofibromatosis type 1, Hirschsprung’s disease, and other neurocristopathies, suggesting a common genetic background (1–3).

This neoplasm originates from neural crest cells derived from the sympathetic nervous system, adrenal medulla, or paraspinal ganglia. The clinical presentation can vary from an asymptomatic mass to symptoms resulting from local invasion and/or distant disease spread. The natural history of neuroblastoma is highly variable, ranging from relatively indolent biological behavior to a high-risk clinical phenotype with a dismal prognosis (1–3).

Age, stage, and biological features found in tumor cells are important prognostic factors for risk stratification and treatment assignment. The multimodal therapy approach includes myeloablative chemotherapy (CTX), radiotherapy (RT), immunotherapy (IMT), and aggressive surgical resection (1–3).

Hyperbaric oxygen therapy (HBOT) has been proposed as a complementary measure to overcome tumor hypoxia, which is considered one of the hallmarks of cancer treatment resistance. Even when isolated, HBOT appears to interfere with tumor growth, metastases, angiogenesis, and anticancer gene expression. Additionally, combining HBOT with specific treatments enhances the production of reactive oxygen species (ROS), inducing apoptosis (4). In this setting, HBOT has been clinically successfully tested with meta-iodobenzylguanidine (^131^I-MIBG) in recurrent neuroblastoma stage IV by combining various biochemical and cellular mechanisms that lead to tumor cell death. Currently, HBOT is considered by the European Committee for Hyperbaric Medicine (ECHM) as a modality of treatment for recurrent neuroblastoma stage IV (degree of recommendation II/level of evidence C) (5).

This article aims to review the relevant literature on the neuroblastoma pathophysiology, clinical presentation, and different biological and genetic profiles, and to discuss its management, focusing on HBOT.

## 2 Pathophysiology

The term neuroblastoma refers to a group of neoplasms of common origin, recognized by James Homer Wright as neuroblastic tumors. The spectrum of neuroblastic tumors is classically classified into three groups with different biological behaviors, reflecting the increasing maturation of sympathetic nervous tissues: ganglioneuromas (benign behavior), ganglioneuroblastomas (intermediate behavior) and neuroblastomas (malignant behavior). More recently, the International Neuroblastoma Pathology Committee (INPC) re-divided it into four categories: neuroblastoma (schwannian stroma-poor tumors), intermixed ganglioneuroblastoma (schwannian stroma-rich tumors), nodular ganglioneuroblastoma, and ganglioneuroma (schwannian stroma-dominant tumors). Each category includes several subtypes according to the degree of cell differentiation (1–3).

Neuroblastoma is an embryonal neuroepithelial malignancy of the sympathetic nervous system arising from neuroblasts (pluripotent sympathetic cells). Under normal conditions, neural crest cell precursors invaginate, migrate along the neuroaxis, and populate the sympathetic ganglia, adrenal medulla, and other sites. However, migration, maturation, or differentiation defects can lead to neuroblastoma formation, typically composed of immature small round blue cells. Thus, the origin and migration pattern of neuroblasts during fetal development explains the anatomic distribution pattern along the peripheral sympathetic nervous system: abdominal cavity (40% adrenal, 25% paraspinal ganglia), chest (15%), pelvis (5%), cervical (5%), miscellaneous (12%), and even occult (1%). This cancer metastasizes via lymphatic and hematogenous dissemination, most commonly in the lymph nodes, bone marrow, cortical bone, and liver (1–3).

Several chromosomal and molecular markers have been extensively investigated in the last decades. Still, data is scarce concerning the genes involved in interrupting normal neuroblast differentiation, their malignant transformation, and neoplastic progression. Concurrently, these biological markers were evaluated to determine their value in predicting prognosis, with some being incorporated into the strategies used for risk stratification (1–3).

In 1983, a gene frequently amplified in cells was identified: the proto-oncogene MYCN (located on the short arm of chromosome 2). The MYCN gene is a member of the MYC family of transcription factors and encodes a protein with a basic helix-loop-helix protein 37, also known as N-myc proto-oncogene protein. In about 20% to 25% of neuroblastomas, MYCN amplification is observed, with consequent activation. Deregulated gene expression was tested in mice, which eventually developed neuroblastomas, proving their involvement in oncogenesis. Patients whose tumors have MYCN amplification tend to have rapid tumor progression and poor prognosis, even with other favorable factors, such as low-stage disease or IVS disease (1–3).

Another interesting finding is an increase in the expression of targeted genes. Furthermore, anaplastic lymphoma kinase (ALK) is highly expressed, mainly in hereditary, and in 5-15% of cases from somatic origin. Essentially, single-base mutations occur in the kinase domain, leading to constitutive activation of the kinase domain and, subsequently, contributing to a premalignant lesion (2). Also, the activation of the ALK oncogene can increase cell proliferation and survival, making it a great candidate for specific biological targeted therapy (3).

The association with other pathologies, such as Hirschsprung’s disease and/or central hypoventilation syndrome, has also demonstrated that loss-of-function mutation may happen in the homeobox gene PHOX2B. Consequently, if the patient has a positive family history of and/or the aforementioned diseases, ALK and PHOX2B mutations should be genetically screened (2).

Moreover, the Children’s Oncology Group (COG) study has concluded that few alleles might contain single-nucleotide-polymorphism mutations that are more present in children than in healthy controls, essentially the ones in the gene FLJ22536 at chromosome 6 long arm position 22.3 (6p22.3), gene BARD1 (BRCA1-associated Ring Domain 1) at 2q35, as well as at 1q21 (2).

The tallying of the genetic mutations involved in the onset and development of neuroblastoma can be divided into two main categories: 1) hyperdiploidy, which contains modifications at a whole chromosome level (correlated with better outcomes); 2) segmental chromosomal changes (amplification of M-YCN, loss-of-heterozygosity in 11q, 1p, gain of function at 17q and activation of ALK) (related to worse prognosis) (1, 2).

## 3 Clinical presentation

As the sympathoadrenal lineage of the neural crest remains the origin, this tumor may arise anywhere in the sympathetic nervous system. Hence, the primary tumor site is within the abdominal cavity, followed by the adrenal medulla. Due to its inherent heterogeneity, the clinical presentation is highly variable and reliant on tumor location.

An infant with an abdominal mass without pain is usually the primary setting of this disease. Most of the symptoms will result from the mass effect of the tumor on surrounding structures such as 1) airway, originating dyspnea; 2) great vessels, leading to ischemia and, subsequently, necrosis; 3) spinal cord, present in 5% of patients, and causing neurological symptoms like paraesthesia/anesthesia, motor muscle weakness or significant pain. Furthermore, systemic non-specific symptoms, like fever, weight loss, and asthenia, may arise (3).

Moreover, metastatic disease is another reason for symptomatic manifestations. The principal metastatic sites include cortical bone, non-contiguous lymph nodes, liver, and bone marrow. This neoplasm frequently spreads to the ostial part of the ocular orbit, originating proptosis and/or periorbital ecchymosis, known as ‘Racoon eyes’ (1). Skin can also be affected by metastatic subcutaneous nodules (3). After bone marrow invasion, patients may present arterial hypertension due to a renin-dependent mechanism, irritability, and intense bone pain (1).

Finally, the paraneoplastic syndrome may also be responsible for symptoms, being more associated with localized disease, thus with a better prognosis. If the mass has a cervical location, Horner syndrome might arise. The two principal paraneoplastic syndromes include 1) increased secretion of vasoactive intestinal peptide, manifesting as refractory aqueous diarrhea; 2) opsoclonus-myoclonus, manifesting as a triad of symptoms that consist of ataxia, irregular muscle movements, and nystagmus (present in 2-4% of patients) (1, 3).

## 4 Diagnosis

The first necessary approach is primary tumor assessment through cross-sectional image exams, such as computed tomography (CT) or magnetic resonance imaging (MRI). Choosing one method over another is based on the inferred location: CT is indicated in the mediastinal, abdominal, or pelvic area. The MRI should be used for the spinal cord (1, 3).

As the staging is the subsequent phase, CT and/or MRI could further evaluate local and distant invasion. The ^131^I-MIBG scintigraphy is also important in detecting bone or soft tissue metastases, being the preferred method due to its enhanced specificity and sensibility. If doubt remains, single photon emission CT (SPECT/CT), 18-fluorodeoxyglucose (FDG) PET/CT, and technetium 99m bone scintigraphy may be indicated (1).

Since bone marrow involvement is relatively frequent, aspirates and, subsequently, biopsies should be performed, after which it is essential to apply immunocytochemical and PCR techniques for the detection of cells and specific transcripts such as PgP9.5, GD2 synthase, and tyrosine hydroxylase (3).

Finally, a biochemical analysis helps to assess the prognosis, specifically the vanillylmandelic acid/homovanillic acid ratio, lactate dehydrogenase, and serum ferritin (3).

## 5 Treatment

Since pathophysiology is not yet fully understood, treatment is highly heterogeneous and relies on the prognostic risk based on tumor resectability, biological, genetic, clinical, and histological features. Overall, it is possible to understand that non-high-risk tumors tend to have reduced-to-non-treatment, contrarily to high-risk neoplasms (1–3).

Patients under 6 months of age, adrenal solid tumors with less than 3.1 cm, or adrenal cystic tumors with less than 5.0 cm only need expectant observation. In patients with stage I from INSS (International Staging System) (6), consisting of “localized tumor with complete gross excision with or without microscopic residual disease,” only surgical resection is required, independently of biological features (3).

For patients with stages IIA and IIB from INSS, who have localized tumor that is not fully resectable but has favorable characteristics, surgery is essential, and CTX is selectively applied if the location is life- or organ-threatening or if the disease is progressive or recurrent (3).

Finally, patients with stage III from INSS, which comprises: “unresectable unilateral tumors across the midline,” and children less than 18 months of age, with stage IV from INSS, which is “any primary tumor with dissemination to distant lymph nodes, bone, bone marrow or other organs” are classified as intermediate risk and need both surgery and CTX (3).

A subset of stage IV is still considered a non-high risk when the patient is less than 18 months old, with a small-sized tumor, metastases restricted to bone marrow (< 10% involvement), liver and skin, and favorable histology and biology (such as the presence of hyperdiploidy and single-copy MYCN). In this subgroup, minimal support therapy is needed, with CTX and surgery only performed in patients with disseminated intravascular coagulation or respiratory distress induced by significant abdominal involvement (3).

The primary challenge in neuroblastoma treatment remains high-risk tumors, namely stage IV from INSS disease with unfavorable characteristics, especially recurrent or relapsed pathology. The main issue regarding this kind of tumor presentation is the occurrence of extensive hypoxic areas that can remarkably increase the resistance capacity to CTX and RT. The high-risk neuroblastoma treatment regimen can be divided into three sections: 1) remission induction, where the goal is to eliminate the tumors at gross; 2) remission consolidation, to avoid tumor regrowth; 3) maintenance, to eradicate minimal residual disease and prevent relapse (2).

In the first phase, the main CTX regimen, introduced by the Memorial Sloan-Kettering Cancer Center, is comprised of 5 cycles of intensive induction treatment (vincristine, doxorubicin, and cyclophosphamide interspersed with cisplatin and etoposide) with a collection of peripheral blood stem cell at the end of 3^rd^ cycle. Subsequently, the patient’s response is evaluated, which may constitute a prognostic biomarker (2, 3).

For the consolidation regimen, platin salt and etoposide are maintained, with additional cyclophosphamide pursued by a combination of thiotepa and cyclophosphamide and local RT. Other therapeutic regimens include single bone marrow transplantation or cyclophosphamide plus thiotepa followed by cyclophosphamide, etoposide, melphalan, or alternatively, busulfan combined with melphalan (3).

At last, aiming to eradicate residual disease and prevent relapse, some targeted therapies are being studied. One of the first approaches for refractory or relapsed high-risk neuroblastoma patients was ^131^I-MIBG therapy. It uses ^131^I-MIBG-targeted radionuclides to achieve selective radioactive treatment exclusively in ^131^I-MIBG-producing cells, essentially tumor cells (3). Recently, a meta-analysis with 26 clinical trials and 883 neuroblastoma patients reported a pooled rate of objective response of 39% and 28% for ^131^I-MIBG monotherapy and in combination with other therapies, respectively. The pooled occurrence rates of thrombocytopenia and neutropenia in ^131^I-MIBG monotherapy studies were 53% and 58%. In a combination regimen, the pooled occurrence rates of thrombocytopenia and neutropenia were 79% and 78% (7). The primary underlying mechanism relies on the augmented expression of noradrenaline transporters, which enhances ^131^I-MIBG intake into tumor cells (7–13).

Furthermore, alternative therapies include retinoids since their capacity to increase the terminal differentiation of neuroblastoma cells has previously been demonstrated. Moreover, as this tumor develops hypoxic areas, angiogenesis inhibitors might decrease neuroblastoma aggressiveness and, consequently, prevent cancer relapse (1).

Another relevant target is disialoganglioside GD2, expressed on the surface of this tumor’s cells. An antibody against GD2 has already been developed in animal models and has shown promising results in eliminating metastatic disease. The chimeric anti-GD2 monoclonal antibody ch 14.18, in combination with granulocyte-macrophage-colony stimulating factor and interleukin-2, revealed an exciting amelioration of 2-year EFS (2).

Moreover, as neuroblastoma is highly heterogeneous and, as a consequence, multiple genetic biomarkers have already been identified (e.g., MYCN, ALK), several research groups are developing antibodies against specific genetic biomarkers to counteract the aggressive replication of neuroblastoma cells, as well as modifying the tumor microenvironment (TME), which is intrinsically associated with tumor growth and immune system evasion (11–13).

Countless therapeutic approaches are being investigated to evade neuroblastoma resistance and relapse. One of the most promising treatments may be HBOT.

## 6 Hyperbaric oxygen therapy

The word “baric” and its prefix “hyper” define the purpose of HBOT, which consists of breathing 100% of O_2_ under hyperbaric conditions, frequently ranging between 2.0 and 2.8 atmospheres absolute (ATA) (14). Its main effect is exerted by the elevation of the inspired gas partial pressure, together with an increase in the fraction of inspired O_2_ (15), resulting in the enhanced amount of O_2_ dissolved in plasma and an increase in the O_2_ delivered to tissues, independently of hemoglobin (4, 16–18).

Hemoglobin is mainly responsible for carrying O_2_ in the blood, with O_2_ saturations of ~95% under normal atmospheric pressure, while 0.32% is dissolved in the plasma (14). In these conditions, the total O_2_ content in the blood is ~21 ml O_2_/dl, with tissues consuming an average of 5 to 6 ml O_2_/dl blood. Because hemoglobin becomes fully saturated with 100% O_2_ at sea level (normobaric pressure), the only way we have to change the blood O_2_ content is to raise its amount dissolved in the plasma (19). Henry’s law states that the amount of dissolved gas in a liquid is directly proportional to the partial pressure of the gas above that liquid. Therefore, breathing 100% O_2_ at 3 ATA means we will have partial pressures higher than 2000 mmHg. Taking into account the solubility factor for O_2_ (0.0024 mL O_2_/dl of blood per mmHg), we can reach at least 4.8 ml O_2_/dl blood dissolved in plasma and a total O_2_ content of 24.8 ml O_2_/dl blood, a net increase of 4.56 ml O_2_/dl blood or 22.5%, thus enhanced the delivery of that gas to tissues (19).

The way that HBOT affects the body depends on direct and indirect physiologic effects and defines its therapeutic use and main clinical applications. The primary effect is the correction of hypoxia as a direct consequence of ischemia or impairment of O_2_ transport to tissues. Hypoxia is a major factor in developing several pathological conditions and is a hallmark in cancer progression today.

## 7 Application of hyperbaric oxygen therapy in neuroblastoma

### 7.1 Tumor hypoxia

Like many other solid tumors, neuroblastoma is mainly driven by genetic alterations that substantially define its progression and malignancy. Nonetheless, recent data revealed that the interplay mechanisms of the TME play a significant role in the progression and metastases of this neoplasm (20).

A common physiologic condition, which can equally be a cause and a driving force to these alterations, is found: hypoxia. This refers to a deficit in O_2_ delivery to organs, tissues, and cells, meaning an imbalance in supply and demand to fulfill the so-called baseline functions necessary for homeostasis (19). That imbalance can be transitory, acute, or chronic depending on underlying causes and physiological conditions. Despite the differences in O_2_ tensions and demands of individual tissues, on average, they consume 5 to 6 ml O_2_/dl blood at rest, necessary to maintain regular metabolic functions. Therefore, we can properly define hypoxia as a condition where tissues fail to receive the usual amount of O_2_, which, if not corrected, leads to several modifications at a cellular level, organ dysfunction, and, ultimately, death (19). The body has several mechanisms to adjust and maintain adequate tissue oxygenation, such as ventilatory rate, cardiac output, stroke volume, dilatation and/or constriction of the capillary bed. Nonetheless, the cellular responses play a significant role, where hypoxia begins to determine the appearance and persistence of the disease (19). At this cellular level, O_2_ is a critical component in energy production, used as the final receptor in the electron transport chain to form adenosine triphosphate. Less O_2_ in the cell means an energy deficit and cell death occurs if it persists too long (19). Additionally, some studies report exposure to hypoxia with an increase in oxidative stress, with low levels of O_2_ favoring the production of ROS and reactive nitrogen species. Both these components have essential roles in hypoxic signaling pathways and their apparent implications in several pathological conditions, particularly cancer development (21).

The concern with tumor hypoxia began in the twentieth century with the work of Mottram JC, who, in 1936 was already concerned with the conditions in which tumor cells could survive, correlating resistance to RT with a reduced O_2_ supply in tumors (22). With scientific progress, it was possible to identify and characterize tumor hypoxia as one of the main features of cancer progression and malignancy (23). The decreased O_2_ levels harm normal and cancer cells, but the latter develop several adaptive strategies to survive and proliferate under hypoxic conditions (24). Höckel M and Vaupel P, in their review of biological and molecular aspects of tumor hypoxia, state that this phenomenon occurs due to poor microcirculation and diffusion conditions (25). In fact, we know today that these structural abnormalities in tumor vasculature disrupt blood flow and widen the distance of O_2_ diffusion from blood vessels, leading to acute and chronic hypoxia in surrounding tissues (26). Contrarily to healthy tissues, tumors are unable to regulate the diminishing O_2_ levels, leading to the development of hypoxia (26).

Despite the type of identified hypoxia, acute (cycling hypoxia) or chronic, both act in tumor cells and the so-called TME, affecting cancer progression, angiogenesis, metastases, and resistance to therapy. Several of the hypoxia-induced pathways are directly correlated with the well-described hallmarks of cancer, namely inducing angiogenesis, resisting cell death, enabling replicative immortality, activating invasion and metastases, evading growth suppressors, and sustaining proliferative signaling (27). If we look closely at one of the principal pathways, which includes the hypoxia-inducible transcription factor 1 (HIF-1), the importance of this physiological condition in cancer biology becomes apparent. In the absence of O_2_, HIF-1 promotes several changes that will affect tumor cells in various ways (28): regulation of genes and control of malignant and metastatic phenotype of cancer cells by enhancing cell survivor through growth factors and inhibition of apoptosis; promotion of tumor growth by angiogenesis and neovascularization, mediated by VEGF and bone marrow-derived endothelial progenitor cells; increase in metastatic activity, particularly via epithelial-to-mesenchymal transition, a process that alters the cell phenotype to acquire mobility and migration capacities; resistance to drug treatments by cellular processes of quiescence, inhibition of apoptosis, controlling autophagy, as well as by the lack of O_2_ required for the CTX cytotoxicity. In addition to the genetic and cellular modifications, hypoxia also affects TME, inducing metabolic and molecular changes in endothelial cells, regulation of inflammatory mediators and growth factors, as well as affecting stromal cells, immune cells, and non-cellular components like extracellular matrix, cytokines and other mediators (27). Considering all those changes and interactions in the biology of cancer promoted by hypoxia, it seems logical that one of the therapeutic targets of cancer, specifically in treating neuroblastoma, should be the correction and overcoming of tumor hypoxia.

Focusing on the HIF family, the primary mechanism of this family consists in the dimerization of the subunits – alpha, which is O_2_-dependent (HIF-1α, HIF-2α, and HIF-3α), and beta, which is O_2_-independent. Under normoxic conditions, the alpha subunits of HIF are usually targeted for ubiquitination by the hydroxylation of prolyl particles and, later, are connected with von Hippel Lindau (VHL) proteins, leading to end-stage HIF alpha subunit degradation. However, under hypoxic conditions, the HIF subunits follow a different pathway. HIF-1α proteins respond acutely to low levels of O_2_ (around 1-2%), while HIF-2 α is only induced in extended periods of hypoxia. Additionally, it appears that the downstream signaling of HIF-1α, HIF-2α, and HIF-3α is similar amongst this family (29, 30).

Interestingly, neuroblastoma cells seem to express enhanced levels of HIF-1α and HIF-2α genetic material, with discrepant meaning. When the expression of HIF-1α is superior to HIF-2α, the patients show a more favorable prognosis with a less aggressive tumor phenotype. However, when the opposite occurs, the tumor has a higher probability of being found in higher stages of development with a subsequent worse prognosis associated (30, 31). Furthermore, it was noticed that clinical neuroblastoma samples seem to present increased concentrations of tumor cells with HIF-2α near blood vessels. This means that HIF-2α can be a biomarker for specific populations of neuroblastoma stem-cell-like and/or neural crest cells. Still, it may also lead to increased production of VEGF and other cytokines responsible for neovascularization, consequently leading to more aggressive phenotypes (30–32).

Therefore, HIF proteins, especially HIF-2α, are presented as potential targeted therapies. Thus, to overcome these hypoxia-induced proteins, there is a need to change the hypoxic TME. One way to accomplish that could be through HBOT.

### 7.2 Hyperbaric oxygen therapy and neuroblastoma

Nonetheless, several doubts arise about whether HBOT would promote the malignant growth of tumors. Suppose HBOT is an adjunctive therapy in wound healing, promoting the proliferation of fibroblasts, epithelial cells, and blood vessels. Could it not have the same effect on malignant tumors (33)?

However, a recent review by Feldmeier J et al. revealed that HBOT not only had no enhancing effect on tumor growth or promoting *de novo* cancer, but it may also have a cancer inhibitory effect as a radiosensitizer (33). Concerns about the possibility of HBOT promoting tumor angiogenesis, similar to the same wound healing process, do not consider the different natures of tumor vasculature and several other pathways for cancer growth. The authors conclude that HBOT administration should not be withheld in patients for whom its benefit is proven, such as delayed radiation-induced injuries, due to concerns that this treatment may cause tumor recurrence or metastases. Additionally, a study about the effect of HBOT on human oral cancer cells, using apoptosis and proliferating cell nuclear antigen to evaluate tumor progression, showed no evidence for the growth or proliferation of cancer cells after HBOT (34).

The HBOT has been studied as a complementary treatment to enhance the effects of RT and CTX, with promising results. As Alpuim Costa D et al. pointed out, “the presence or absence of molecular O_2_ dramatically influences the biological effect of radiation exposure” (4). Already by 1953, Gray LH et al. showed that the sensitivity of tumor cells to X-rays was about three times greater when irradiated in a well-oxygenated environment when compared to low concentrations of O_2_; they also concluded that the effectiveness of RT was likely to increase if the patients were breathing O_2_ at the time of irradiation (35). The same authors proved that the O_2_ concentration influences the radiosensitivity of tumor cells at the time of irradiation (35). In the absence of O_2_, the radiation dose needed to achieve the same biological effect is three times higher (36–38). Ionizing radiation damages DNA directly and indirectly. Indirect damage occurs through radiation formation of ROS, which is O_2_ dependent. When water molecules are exposed to ionizing radiation, they undergo radiolysis, forming hydroxyl and hydrogen radicals. Hydrogen radicals react with O_2_, producing perhydroxyl radicals, causing irreversible damage to DNA. The TME with high O_2_ levels leads to increased production of ROS and, consequently, to a higher effect of radiation. In 2007, Overgaard found that among several modified methods of hypoxemia to overcome tumor hypoxia, HBOT had the most pronounced effect (39). The efficacy of HBOT in reducing tumor radioresistance has been demonstrated in experimental studies and clinical trials (40, 41). Moreover, HBOT at 2 ATA increased the effectiveness of RT in head and neck tumors and achieved promising results in the local control of high-grade gliomas (41, 42). Additionally, in a pilot study by Dowling S et al. HBOT was safely used with a perfluorocarbon-based solution (Fluosol DA 20%) combined with RT (43).

From another integrative perspective, HBOT and the ketogenic diet (KD) were explored as non-toxic therapies that exploit overlapping metabolic deficiencies of cancer. Cancer cells often exhibit a distinctive metabolic preference for glucose, a phenomenon known as the Warburg Effect or aerobic glycolysis. Cancer cells heavily rely on glycolysis to convert glucose into energy, unlike normal cells, even when oxygen is readily available. This distinct metabolic behavior leads to cancer cells having a significantly higher glucose uptake than normal cells. The KD is a low-carbohydrate, high-fat diet that decreases blood glucose and elevates blood ketones, which can be used as an alternative source. Abnormal tumor vasculature creates hypoxic regions that promote cancer progression and further increase the glycolytic dependency of cancers. Some animal and human experiments have explored the KD’s potential to hinder cancer cells’ survival by restricting their access to glucose. The rationale for this emerging combination is that while the KD limits glucose availability for cancer cells, HBOT increases oxygen availability. This strategy could create a hostile environment for cancer cells. Promising preclinical research supports this idea, but further rigorous clinical studies are essential to determine the effectiveness and safety of this approach in cancer treatment (4, 44–47).

#### 7.2.1 Hyperbaric oxygen therapy combined with ^131^I-meta-iodobenzylguanidine

The indication for ^131^I-MIBG therapy in neuroblastoma is established in the *European Association of Nuclear Medicine procedure Guidelines* (48), being indicated for stage II or IV neuroblastoma in lesions with adequate uptake and retention of ^131^I-MIBG in pretherapy ^123^I/^131^I-MIBG scintigraphy. If ^131^I-MIBG does not accumulate in primary neuroblastoma lesions, it should not be used as therapy.

The ^131^I-MIBG is an aralkylguanidine formed by combining the benzyl group of bretylium and guanidine group of guanethidine. This drug is structurally similar to the neurotransmitter noradrenaline. Its uptake by tumor cells is derived from the primitive neural pathway through an active noradrenaline transport system (uptake-1) or passive diffusion. After absorption by cells, it is stored in the cytoplasm and neurosecretory granules, not being metabolized by enzymes. The MIBG labeled with ^131^I is used as a radiotherapeutic agent in neuroectodermal tumors. Decomposition of decaying ^131^I radionuclide results in the emission of ionizing radiation, which has an average tissue range of about 0.5 mm. It was first used in treating neuroblastoma in 1986, and since then, several clinical trials have been developed, such as using ^131^I-MIBG combined with HBOT.

In 1995, Voûte PA et al. compared two groups of patients with recurrent stage IV neuroblastoma after CTX treatment (49). One group was treated with ^131^I-MIBG (n= 36), and another with ^131^I-MIBG combined with HBOT (n= 27). In both groups, ^131^I-MIBG was administered at a dose of 200 mCu in the first treatment and 100 mCu in the subsequent ones. In the HBOT combination therapy group, HBOT sessions were performed 2 to 4 days after treatment with ^131^I-MIBG, 4 to 5 consecutive days in multiplace hyperbaric chambers. The HBOT sessions profile included 12 minutes of pressuring the chamber from 1 to 3 ATA, followed by 75 min at 3 ATA. The decompression profile applied was from the *Canadian Forces Diving Manual* decompression tables. The O_2_ was administered through a nasal/mouth mask, guided by transcutaneous optical O_2_ tension to a target of 1000-1200 mmHg. The 28-month OS of patients undergoing ^131^I-MIBG alone was 12%, whereas that of patients receiving combination therapy was 32%. Furthermore, HBOT was tolerated by all patients with no discomfort or increased toxicity.

**Figure 1:**
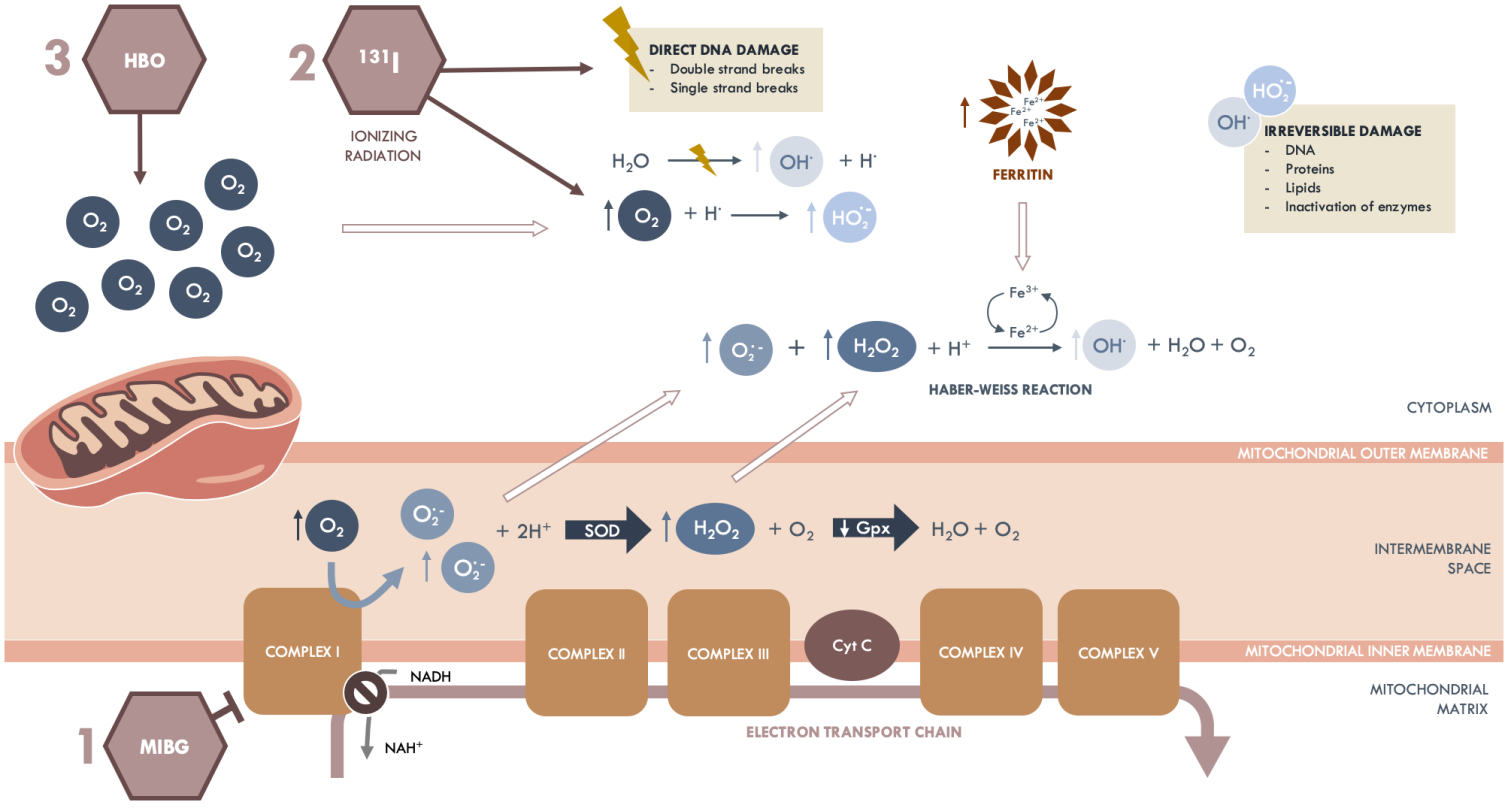
Effects of ^131^I-MIBG combined with HBOT on neuroblastoma cells. The ^131^I-MIBG has an antiproliferative effect by inhibiting complex I of the mitochondrial respiratory chain, leading to increased production of superoxide radicals. In neuroblastoma cells, the activity of H_2_O_2_ detoxifying enzymes is reduced, meaning that superoxide radicals are not converted into hydrogen peroxide and, consequently, into water and O_2_. In the presence of superoxide radicals, hydrogen peroxide is converted to hydroxyl radicals in the iron-catalyzed *Haber-Weiss* reaction, leading to proteins, lipids, and DNA peroxidation. The ^131^I emits ionizing radiation through the decay of the ^131^I radionuclide, directly and indirectly affecting DNA damage. The indirect effect occurs through the formation of ROS by radiolysis of the water molecule, dependent on O_2_. In turn, HBOT potentiates the effect of ^131^I by increasing tumor O_2_ levels. Cyt C, Cytochrome complex; Gpx, Glutathione peroxidase; HBOT, Hyperbaric oxygen therapy; HO_2_ **^·^**, Perhydroxyl radicals; H_2_O, Water molecule; H_2_O_2_, Hydrogen peroxide; ^131^I-MIBG, Meta-iodobenzylguanidine; OH**^·^, ** Hydroxyl radical; O_2_, Oxygen; O_2_ **^·^** ^-^, Superoxide radical; ROS, Reactive oxygen species; SOD, Superoxide dismutase.

The effectiveness of ^131^I-MIBG combined with HBOT seems to be explained by the characteristics of neuroblastoma cells, as they have high levels of ferritin deposits and decreased activity of H_2_O_2_-detoxifying enzymes (catalase and glutathione peroxidase). The ^131^I-MIBG itself is an inhibitor of complex I (NADH-ubiquinone-oxidoreductase), present in the mitochondrial electron transport chain, causing a leak of paired electrons, leading to increased production of superoxide radicals. Under normal conditions, superoxide radicals would be converted into hydrogen peroxide by the superoxide dismutase and, consequently, into water and O_2_ by the catalase. As in neuroblastoma, the activity of the H_2_O_2_-detoxifying enzyme is reduced, and hydrogen peroxide accumulates intracellularly. In the presence of superoxide, hydrogen peroxide is converted into highly reactive hydroxyl radicals (conventionally named ROS) in the iron-catalyzed *Haber-Weiss* reaction, leading to the peroxidation of proteins, lipids, and DNA (50). In addition, the radiation effect is potentiated by HBOT, increasing the intracellular production of NOS and consequent increase in tumor cell damage (**Figure 1**).

## 8 Future perspectives

In this review, we sought to highlight the role and importance of HBOT as an adjuvant to some of the available neuroblastoma treatments, focusing on hypoxia and its role in oncobiology. It is clear that hypoxia acts as a trigger to several signaling pathways that regulate cancer growth. Still, it also induces significant changes in the TME (28) and, as such, should be considered for new treatment strategies.

As Borriello L et al. indicate, neuroblastoma is “truly a disease of the seed and the soil,” in which the seeds can be identified as the neuroblastoma cells and the soil as the surrounding TME (20). The main question is to know to what extent the genetic modifications of these “seeds” can modify the “soil” to maintain its development and survival (20). This disease is driven not only by genetic events but also by the complex interplay between them and their influence on the TME through paracrine mechanisms, which significantly contribute to tumor progression and aggressiveness (20).

Stages III and IV of neuroblastoma represent some of the most difficult solid tumors to treat, and despite intensive research, progress toward a cure remains elusive (51). The main concern regarding high-risk, relapsed, metastatic, and/or resistant neuroblastoma is the increased tumor toxicity of aggressive cancer treatment and the enhanced risk of secondary tumors over the years. One of the strategies to reduce toxicity is to apply the respective treatment locally through nanotechnology. Both CTX and biologically targeted therapy or IMT may be more effective and have fewer side effects due to nanoparticle delivery (51).

Currently, the main treatments available can be summarized as follows: surgery, RT, CTX, facilitation of CTX via nanotechnology, stem cell rescue and myeloablation therapy, ^131^I-MIBG, and IMT. Each of these approaches has its pros and cons. Particularly, multimodal therapeutic strategies and the resulting high toxicity and increased development of secondary malignancies after treatment must be carefully considered (51). Of the latter, IMT holds the most promise in controlling cancer progression, but significant challenges remain, most notably the several immune escape mechanisms employed by neuroblastoma cells (51).

Surprisingly, the combination of HBOT with IMT shows promise in increasing the efficiency of IMT, as HBOT can stimulate the distribution of programmed death-(ligand)1 checkpoint blockade, as well as the activity of tumor-infiltrated lymphocytes through extracellular matrix modulation. Furthermore, HBOT can reduce immunosuppression resulting from hypoxia, enhancing CD4+ lymphocyte activity (52).

Nanotechnology can potentially improve antitumor efficacy and reduce side effects, increasing patients’ quality of life. However, translating from the bench to the bedside process is more complex than it might appear at first glance due to several factors, including mimicking the hypoxic TME (53).

Regarding early diagnosis and treatment monitoring, one of the areas of most significant interest is the evaluation of circulating tumor cells (CTC) and tumor DNA (ctDNA). Due to its discrimination ability to distinguish CTCs/ctDNA from primary tumors and distant metastases (by analyzing their chromosomal aberrations and/or DNA mutations), this technique is particularly relevant. Compared to adult solid tumors, neuroblastoma has fewer mutations, which presents a unique advantage. Moreover, it is possible to use single-cell and CTC proteomics analysis, which may lead us to a new step attributable to the capacity to detect cells more likely to resist treatment (54).

As cancer therapies keep evolving, incredible novelties, such as CAR-T cells, need to be highlighted, where several clinical trials in high-risk neuroblastoma are underway to evaluate this therapy´s potential. Yet, CAR-T cells in solid tumors do not seem to have a stellar impact, which the suppressive TME might explain. Therefore, there is a definite need to improve knowledge of TME in neuroblastoma to prevent immune system escape and increase the effectiveness of this therapy (55).

Hereafter, we expect that the investigation and understanding of these intricate interactions will undoubtedly guide new perspectives in clinical studies on neuroblastoma.

## Author contributions

DAC: Conceptualization, Data curation, Formal Analysis, Investigation, Methodology, Software, Supervision, Validation, Writing – original draft, Writing – review & editing. JGN: Investigation, Validation, Writing – original draft, Writing – review & editing. MSA: Data curation, Methodology, Software, Validation, Writing – original draft, Writing – review & editing. NG: Investigation, Validation, Writing – original draft, Writing – review & editing. JAR: Data curation, Methodology, Software, Validation, Writing – original draft, Writing – review & editing. CEA: Supervision, Writing – review & editing.
